# Effect of steam de-alumination on the interactions of propene with H-ZSM-5 zeolites†

**DOI:** 10.1039/d0ra03871g

**Published:** 2020-06-17

**Authors:** Alexander P. Hawkins, Andrea Zachariou, Stewart F. Parker, Paul Collier, Nathan Barrow, Ian P. Silverwood, Russell F. Howe, David Lennon

**Affiliations:** School of Chemistry, University of Glasgow Joseph Black Building Glasgow G12 8QQ UK David.Lennon@glasgow.ac.uk; UK Catalysis Hub, Research Complex at Harwell, STFC Rutherford Appleton Laboratory Chilton Oxon OX11 0FA UK; ISIS Neutron and Muon Source, STFC Rutherford Appleton Laboratory Chilton Oxon OX11 0QX UK; Johnson Matthey Technology Centre Blounts Court, Sonning Common Reading RG4 9NH UK; Department of Chemistry, University of Aberdeen Aberdeen AB24 3UE UK

## Abstract

Steam de-alumination is used to prepare a H-ZSM-5 material representative of industrial acid zeolite catalysts. Characterisation shows extensive loss of zeolite acidity but minimal loss of framework crystallinity in the treated material. The material's interaction with propene is probed by means of inelastic and quasielastic neutron scattering, providing information on the reactivity and mobility of the propene respectively. These results are compared to those previously obtained for propene in the untreated zeolite. The steaming treatment resulted in decreased reactivity of the zeolite toward olefin oligomerization, higher temperatures for reaction initiation, and increased mobility of the propene in the zeolite at all temperatures. Analysis of the motions of the propene revealed by QENS shows the mobility to be comparable to those previously reported for propane in similar materials but occurring at slower velocities due to the greater rigidity and polarisation of the propene molecule.

## Abstract 2

Inelastic and quasi-elastic neutron scattering are used to investigate how steaming changes the physico-chemical characteristics of the zeolite ZSM-5.

## Graphical abstract

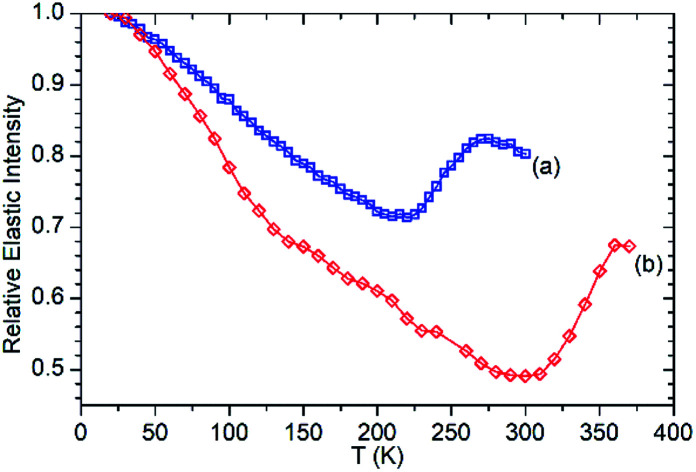

## Introduction

Propene is an important component in the overall petrochemical supply chain. It is targeted as a desired product molecule in the cracking of heavy hydrocarbons through selective fluidised catalytic cracking (FCC) due to its value as a platform chemical.^1–4^ It may be used as a precursor for the formation of clean synthetic fuels and high quality lubricating oils through use of selective oligomerization reactions.^5–7^ Propene can be produced from alternative sources through the methanol-to-olefins reaction,^8,9^ and may play an important role as a key intermediate in the formation of the initial C–C bonds and the hydrocarbon pool in the methanol-to-hydrocarbons (MTH) process.^10^

All of these applications make use of ZSM-5 type acid zeolites as catalysts for the hydrocarbon conversion reactions.^11,12^ Acid zeolites are microporous aluminosilicate materials with internal acid sites, with each acid group offsetting the charge from and being associated with a Si → Al substitution within the overall tetrahedral SiO_2_ framework. They are selective catalysts due to shape-selectivity effects arising from the pore structure. The internal acid sites can catalyse both the formation and cleavage of C–C bonds as well as isomerisations, with the dominating reactions dependant on reaction conditions. The product of these reactions is then influenced by the presence of the pore walls, which favour certain products or reaction routes and disfavour others due to steric constraints around the active sites. The MFI-type structure of ZSM-5 is particularly favourable to reactions involving gasoline-range hydrocarbons and light olefins such as propene. The product composition is a statistical mixture of the various possible products, with the sterically favoured ones predominating. The nature of the zeolite–propene interactions and how propene diffuses through the MFI microstructure are therefore key to understanding the catalytic activity of ZSM-5 since this is what dictates the relative probability of a given species being released as a product. Further reactions or isomerisations within the zeolite may lead to the formation of a molecule which is too large to move freely through the network leading to pore blockage and eventual catalyst deactivation.^6,13^ However, while the zeolite's acid site density and the mean diffusion path within the zeolite are the most important contributors to the propene–zeolite interactions, these properties do not remain constant throughout the catalyst's useful lifetime. Hydrothermal conditions in catalytic use result in partial de-alumination of the zeolite framework with a corresponding reduction in the number of acid sites, since removing an aluminium atom also removes its associated Brønsted O–H acid group, and increases mesoporosity due to removal of portions of the framework.^14^ The rate of de-alumination is first order with respect to the number of aluminium substitutions within the zeolite, meaning that loss of acidity is initially rapid before the catalyst achieves a pseudo steady state level of acidity, which it occupies for the majority of its active lifetime.^15^ Effects of this loss of acidity can be dramatic, with numerous studies reporting improved selectivity, stability and even activity in steamed zeolites relative to studies performed using fresh materials.^13,16,17^ Knowledge of how these framework changes affect fundamental properties like propene diffusion and acid site bonding in the zeolite is therefore key to improved understanding of the catalytic activity and the mechanism behind these improvements. Changes due to de-alumination from catalytic use can be simulated by the steam treatment of fresh zeolite, allowing the generation of model zeolite materials whose properties closely match those of used or partially-deactivated catalysts.^14,15,18^

The low temperature oligomersiation of olefins in HZSM-5 has been previously studied by ^13^C NMR spectroscopy^19^ and infrared spectroscopy.^20–22^ These studies agree that at low temperatures olefins such as ethene and propene readily form oligomeric species, and that the rates of oligomer formation vary with the Al content of the zeolite^21^ and the distribution of acid sites within the zeolite crystals.^22^

The use of neutron techniques offers several advantages to the study of zeolite–catalysed systems. Neutron spectroscopic methods which are used in the study of catalysis are based on the scattering of neutrons from the atomic nuclei of the sample *via* the strong nuclear force.^23^ This means that the intensity of scattering is determined by the scattering cross section of the elements involved in a given mode, which is a property of the nucleus itself and is therefore both element and isotope dependant. Neutron spectroscopic techniques often make use of the inelastic incoherent scattering cross section, which for ^1^H is forty times larger than that of any other atom present.^24^ For vibrational studies using the inelastic neutron scattering (INS) technique, this means that hydrogenous modes dominate the spectrum, allowing the modes of interest to the catalytic activity of the zeolite and its interactions with adsorbed hydrocarbons to be studied with minimal interference from the zeolite framework. This is particularly important since it allows unhindered observation of the C–H and C–C–H deformations which lie below 2000 cm^−1^ and are masked by strong Si–O and Al–O framework modes in infrared spectroscopy and high levels of fluorescence in Raman studies. Additionally, the highly penetrating nature of neutrons, due to only interacting with atomic nuclei, means that neutron techniques are a bulk probe which can observe species even at the centre of larger zeolite crystallites, while the lack of selection rules means that INS can in principle observe all vibrational modes in the spectrum of the system. Several reviews have highlighted the advantages of neutron techniques in this regard,^25–27^ and they have been successfully applied to the study of several zeolite-based catalytic systems.^28–32^

In addition to the information on zeolite–hydrocarbon bonding interactions available through INS, neutron scattering offers the possibility of obtaining information on the diffusive motions of the hydrocarbons through the quasi-elastic neutron scattering (QENS) technique. QENS measures the scattering from atoms in motion and provides information on motions occurring across Ångstrom length scales and timescales from picoseconds to nanoseconds depending on instrumental design. These values correspond closely to the length and time scales of the confined diffusion in zeolites, and the use of QENS in the study of such systems is well established, particularly for the motions of adsorbed alkanes.^33–40^

QENS of hydrogen-containing materials is also dominated by the incoherent scattering of ^1^H and, therefore, by the hydrogen contributions in zeolite–hydrocarbon systems. In QENS it is necessary to consider the scattering intensity with respect to both energy transfer (Δ*ω*, meV) and momentum transfer (*Q*, Å^−1^). These are measured together as the incoherent scattering function *S*(*Q*,*ω*), which is the time and space Fourier transform of the van Hove autocorrelation function. It therefore contains all the information on the dynamics of the atoms in the sample, although the aforementioned dominance of the hydrogen contributions means that only motions that involve hydrogen movement are generally resolvable. Due to the low energy values associated with translational and rotational motions, the incoherent scattering function takes the form of a broadening of the peak due to elastic scattering from the sample at Δ*ω* = 0. Although, theoretically, the elastic scattering takes the form of a delta function, any instrument will have a finite energy resolution which will also contribute to the peak broadening. The measured scattering function will therefore be a convolution of the scattering due to elastic and quasielastic contributions with the instrument resolution function. Experimentally, both the elastic scattering and instrument resolution functions can be combined and modelled by use of a scaled spectrum of the sample collected at low temperatures where the motion in the sample is assumed to be zero. This allows the collected experimental scattering function to be modelled by the equation

1 *S*(*Q*,*ω*) = *S*^quasi^(*Q*,*ω*) ⊗ *R*(*Q*,*ω*) + *B*(*Q*,*ω*)

where *R*(*Q*,*ω*) is the resolution function and *B*(*Q*,*ω*) represents a linear background which embodies the contributions from motions which are too fast for the instrument to observe.^40,41^

Fitting the experimental data to eqn (1) allows the extraction of the quasielastic contribution to the scattering, which takes the form of one or more Lorentzian functions with each function corresponding to an individual molecular motion which is resolvable in the collected data. The way the half-width at half-maximum (HWHM, *Γ*) of these Lorentzians varies with *Q* is characteristic of the type of motion the Lorentzian embodies, since they ultimately derive from the van Hove autocorrelation function for the motion. For the simplest case, that of Fickian diffusion, this relationship is linear with respect to *Q*^2^, given by *Γ* = *D*_s_*Q*^2^ where *D*_s_ is the self-diffusion coefficient. More complex motions such as jump diffusion introduce deviations from this linear relationship at higher *Q* values, which correspond to shorter length scales, allowing determination of the dynamics of the motion which may be detected through examination of the observed *Γ vs. Q* relationships in the fitted data.^40^

The benefits of the QENS and INS techniques described above mean that they are ideally placed to answer questions about the changes in fundamental zeolite–hydrocarbon interactions due to zeolite de-alumination, and the results reported here seek to apply these advantages to the ZSM-5/propene oligomerization system at low temperatures. The preparation of the steamed zeolite material, its characterisation and the comparison of its properties and interactions with those of the fresh zeolite material is also described. As far as we are aware, this is the first use of QENS to examine the movement of adsorbed hydrocarbons in a partially de-aluminated zeolite with properties more representative of a late-lifetime zeolite catalyst.

## Experimental

The ZSM-5 used was a powder form commercial material grade H-ZSM-5 zeolite supplied by Johnson Matthey and calcined in air for 12 hours at 773 K to remove the residual synthesis template; this will henceforth be referred to as the catalyst. Characterisation of this fresh catalyst, henceforth referred to as ZSM-5-FR, showed it to possess a Si : Al ratio of 30 : 1, established by SS-NMR. Previous investigations using the same catalyst have shown it to possess an average crystallite size of 0.1 × 0.1 × 0.5 μm:^42^ the calcined catalyst was sieved to provide consistent crystallite agglomerate sizes in the 200–500 μm range.

Steam treatment of the fresh catalyst to generate the artificially aged material for this investigation was carried out at the ISIS facility's sample preparation laboratories. 15 g batches of the fresh zeolite were loaded into an Inconel reaction vessel equipped with gas-handling fittings and mounted on a gas handling reaction rig; a full technical description of this apparatus is available elsewhere.^43^ The reactor was heated to 873 K under flowing helium (100 cm^3^ min^−1^, BOC, >99.999%) at a rate of 5 K min^−1^. Once at temperature, steaming was initiated by the introduction of 1 g g_cat_^−1^ h^−1^ of deionised water into the inlet gas stream using a HPLC pump (Teledyne, model M1010SNN1C), with the positioning of the gas inlet at the bottom of the reactor ensuring full vapourization of the water feed prior to contact with the zeolite bed. Steam treatment was carried out for 48 hours, with these conditions being chosen to generate a de-aluminated end product.^14,18^ Following water shutoff, the temperature of the reactor was reduced to 623 K and helium flow maintained for a further 6 hours to remove all adsorbed water from the sample. The reactor was then sealed using the integrated fittings, cooled to room temperature and transferred to an argon glovebox (MBraun UniLab MB-20-G, [H_2_O] < 1 ppm, [O_2_] < 1 ppm) to permit preparation of the experimental samples without contamination from atmospheric water. This material will be referred to as ZSM-5-ST.

Characterisation measurements of both ZSM-5-FR and ZSM-5-ST were carried out in order to assess the effects of the steam treatment. Surface area analysis was performed using a Quantachrome Quadrasorb EVO/SI gas adsorption instrument. 0.15 g samples of the zeolites were degassed to <20 mTorr at 523 K and gas adsorption and desorption isotherms were collected across a relative pressure (*P*/*P*_0_) range from 5 × 10^−4^–0.99 using liquid nitrogen as the coolant and N_2_ as the adsorbant gas. Isotherm analysis was carried out using the method of Brunauer, Emmett and Teller using the software supplied with the instrument.^44^ Sample microporosity levels were estimated using the t-plot method of de Boer.^45^ The experiment was repeated three times for each material with the mean and standard deviation values reported.

The degree of framework de-alumination in the sample was determined by ^27^Al NMR. SS-NMR spectra were acquired at a static magnetic field strength of 9.4 T (*ν*_0_(^1^H) = 400 MHz) on a Bruker Avance III console using TopSpin 3.5 software. A widebore Bruker 4 mm MAS probe was used, tuned to 104.27 MHz and referenced to yttrium aluminium garnet at 0.0 ppm. The zeolites were left in a humid environment overnight and packed into zirconia MAS rotors with Kel-F caps. Before and after weight measurements provided the sample mass for normalisation. The rotors were spun at 14 kHz using room-temperature purified compressed air. Nutation tip angle was 22.5°. Relaxation times were 0.1 s. 8192 scans were acquired using a one pulse acquisition program.

To observe the effect of the de-alumination on the acid sites in the zeolite samples, both zeolites were analysed by Diffuse-Reflectance Infrared Fourier Transform Spectroscopy (DRIFTS) using an Agilent Carey 680 FTIR spectrometer equipped with a Harrick praying mantis beam accessory and heated sample cell with gas flow capability. Spectra were collected from 4000–700 cm^−1^ using a liquid nitrogen cooled MCT detector with a spectrometer resolution of 4 cm^−1^ and averaged over 64 scans per spectrum. Samples were purged with dry N_2_ from a liquid nitrogen boil-off source (25 mL min^−1^) then heated to 423 K at 5 K min^−1^ and held for 30 minutes under continued flow prior to spectrum collection to ensure that they represented the dry zeolite in all cases.

Quantification of the changes in acid site population was achieved by means of ammonia temperature-programmed desorption. TPD experiments were carried out using a Quantachrome ChemBET Pulsar instrument equipped with a thermal conductivity detector. Samples were dried at 623 K under flowing helium (15 mL min^−1^) then cooled to 373 K and saturated with ammonia by passing 10% NH_3_ in He (15 mL min^−1^) through the sample for 15 minutes. The sample was returned to helium flow and purged for 2 hours at the same temperature; these conditions being reported to remove any physisorbed ammonia from the zeolite pore network leaving only molecules chemisorbed to Lewis silanol or Brønsted acid sites.^46^ Desorption was then carried out from 373–973 K with a heating rate of 5 K min^−1^ and a 30 minute hold at the highest temperature to ensure full removal of all ammonia.

INS-based investigations of the zeolite–olefin interactions were carried out at the ISIS facility using the TOSCA indirect geometry neutron spectrometer: this instrument is optimised for observation of vibrational frequencies in the 0–4000 cm^−1^ range with best resolution at energies below 2000 cm^−1^, allowing observation of hydrocarbon deformation modes.^47^ An 8.6 g sample of ZSM-5-ST was loaded into an aluminium gas handling sample cell optimised for INS measurements. This cell design is equipped with gas inlet ports and top- and bottom-mounted cell heaters with independent thermocouples to allow for accurate sample temperature control. The portion of the cell which contains the sample forms a 50 × 50 × 10 mm flat plate and makes use of the full area of the TOSCA incident neutron beam. The loaded sample was connected to a gas handling rig, inserted into TOSCA and cooled to the <15 K temperatures suitable for INS data collection using the closed cycle refrigerator (CCR) which forms part of the TOSCA sample environment. A background spectrum of the unloaded ZSM-5-ST was then collected.

Following background collection, the sample was removed from TOSCA and the cell plus associated gas pipework allowed to warm to room temperature. The cell was then cooled to 170 K and loaded with 7.11 × 10^−3^ mol of propene, equating to a loading of 4.8 propene molecules per unit cell: this method has been shown to allow the propene to enter the zeolite while preventing any oligomerization reactions from occurring, even in the highly active fresh catalyst.^42,48^ Specifics of the loading procedure followed are available in the literature.^48^ The sample was returned to TOSCA and a post-loading spectrum was collected. It was then sequentially heated to higher temperatures in the 220–325 K range in order to investigate how the zeolite–propene interactions and reactivity vary with temperature. For each temperature investigated, the sample was heated to the target temperature using the cell mounted heaters, maintained at that temperature for 60 minutes to allow any reactions to run to completion then returned to <15 K for INS measurement. Spectra were collected after heating to 220, 260, 270, 280, 290, 300 and 325 K. The raw neutron time-of-flight data from all experiments was reduced to the energy transfer spectra presented below using the Mantid software package.^49^

QENS measurements to quantify sample mobility were performed at ISIS on the backscattering indirect geometry spectrometer OSIRIS: when used with the high resolution (002) reflection of its graphite analyser crystal this instrument provides an energy resolution of 25.4 μeV and an accessible momentum transfer range of 0.18–1.8 Å^−1^.^50^ 3.3 g of ZSM-5-ST was loaded into a 25 mm diameter aluminium QENS sample can with a 2 mm annulus. This sample mass and cell geometry was chosen to achieve optimal results for QENS analysis, which requires the scattering of approximately 10% of the incident neutron intensity and a uniform sample geometry with respect to the instrument's circular detector bank. This cell design has a single top-mounted gas inlet port and retains the top- and bottom-mounted cell heaters and thermocouples. The sample was attached to a heated line and gas handling panel to allow insertion of gaseous adsorbates while *in situ* and inserted into the OSIRIS CCR. Once installed, the sample was cooled to base temperature (<15 K) and a high signal-to-noise ratio QENS spectrum collected to provide a resolution function for the instrument for use in later data fitting analysis. The sample was subsequently heated to 170, 220 and 270 K and spectra collected at these temperatures to allow subtraction of the zeolite contributions to the combined spectrum following loading. These temperatures were chosen to allow observation of the propene mobility across the maximum possible range of temperatures while remaining below the lowest temperature at which oligomerization was observed in the vibrational dataset collected on TOSCA as described above.

Loading of the QENS sample was achieved by returning the sample cell to 200 K while inside the CCR environment. The zeolite was then dosed with 2.92 × 10^−3^ moles (5.06 molecules per unit cell) of propene using the same method previously used on TOSCA except for the addition of the use of a heated capillary line to allow insertion *in situ*. After 1 hour for diffusion of the propene the sample was cooled to base temperature and a resolution function of the loaded zeolite obtained. A series of short (20 minutes) QENS spectra were then collected at 10 K intervals from 20–370 K to allow quantification of changes in the system's mobility with temperature. 20 minutes of stabilisation time was allocated for any reactions to occur at each temperature, and the spectra at 170, 220 and 270 K were extended to four hours to provide high resolution datasets for fitting analysis in combination with the empty spectra previously collected. Time-of-flight data were reduced using Mantid which was also used to perform the elastic window scan analysis presented in Fig. 6. Background subtraction and peak fitting analysis of the reduced high-resolution spectra was carried out using the DAVE QENS analysis software suite.^51^

## Results and discussion

**Fig. 1:**
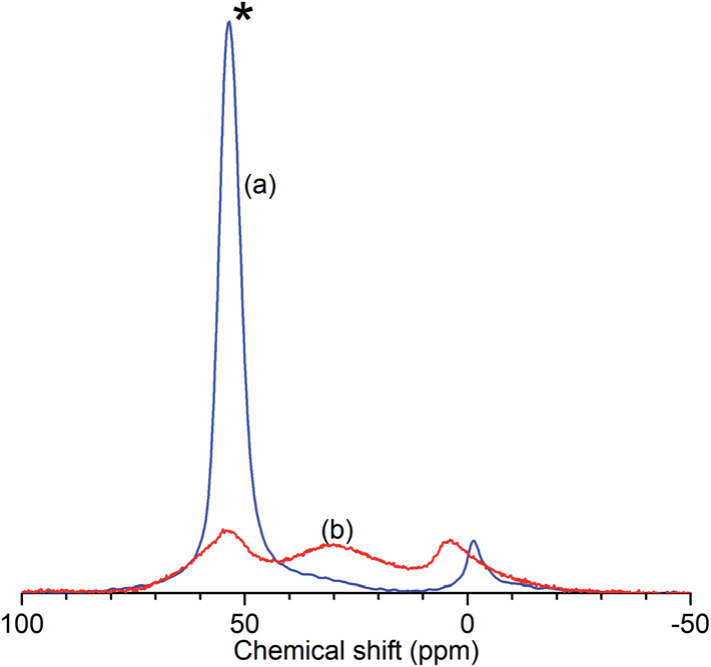
Normalised ^27^Al solid-state MAS NMR spectra of ZSM-5-FR (a) and ZSM-5-ST (b) showing reduced intensity of the AlO_4_ peak (*) due to loss of framework aluminium in the zeolite.

The results of the characterisation analyses are shown in Table 1 and confirm that the steam treatment resulted in extensive de-alumination of the treated zeolite framework. As measured by ^27^Al NMR (Fig. 1), the spectrum of ZSM-5-FR shows a signal for the 4-coordinated AlO_4_ species which constitute the framework aluminium located at 53.4 ppm. An additional peak observed at −1.2 ppm, indicates that the fresh catalyst already contains a small population of octahedral extra-framework aluminium (EFAl) species.^14,52^ Fitting of the NMR data to a Lorentzian function for each peak across the range −50 to 100 ppm was carried out in order to determine the peak intensities at each shift value and the overall intensity in each sample. In the spectrum for ZSM-5-ST the AlO_4_ peak shows a reduction in intensity of 77% relative to the that for ZSM-5-FR, representing a corresponding reduction in the number of framework aluminium nuclei. While the ZSM-5-ST NMR spectrum shows an increase in the intensity of the AlO_6_ peak it is also able to detect an additional, broad peak centred at 30 ppm; although the exact species responsible for this signal is a matter of debate, it is accepted that it represents an environment intermediate between the framework and EFAl states.^15^ Re-fitting of the ZSM-5-FR spectrum with the presence of this peak reveals it to be present at just-detectable levels in the pre-treatment material as well. However, even accounting for the contributions of all these environments there is a reduction of 35% in the overall signal intensity of ZSM-5-ST relative to the fresh material. Since EFAl species are not reported to be mobile enough to leave the zeolite framework without additional treatment,^14^ it can be surmised that the additional EFAl generated by the steam de-alumination process remains present in the sample in a number of different environments, a proportion of which are subject to second-order quadrupolar interactions which render them invisible to NMR detection despite the high MAS frequency employed. Generally, this ‘NMR-invisible’ fraction of the EFAl is attributed to the presence of clustered aluminium species containing multiple nuclei. The fit parameters and derived peak areas used in the analysis of the NMR data are reproduced as Table S1 in the ESI.†

**Table 1:** Zeolite parameters before and after steam treatment as established by ^27^Al NMR, ammonia TPD and BET surface area analysis

Sample	^27^Al NMR		Ammonia TPD				BET surface area	
	Total relative intensity (±0.03)	AlO_4_ relative intensity (±0.03)	Volume adsorbed (μmol_NH_3__ g_ZSM-5_^−1^)		Population (sites per unit cell)		Micropore surface area (m^2^ g^−1^)	Total surface area (m^2^ g^−1^)
			Silanol	Brønsted	Silanol	Brønsted		
ZSM-5-FR	1.00	0.91	434 ± 43	366 ± 35	2.62 ± 0.26	2.22 ± 0.21	232 ± 9	370 ± 11
ZSM-5-ST	0.65	0.21	34 ± 8	19 ± 3	0.21 ± 0.05	0.12 ± 0.02	218 ± 5	357 ± 22

**Fig. 2:**
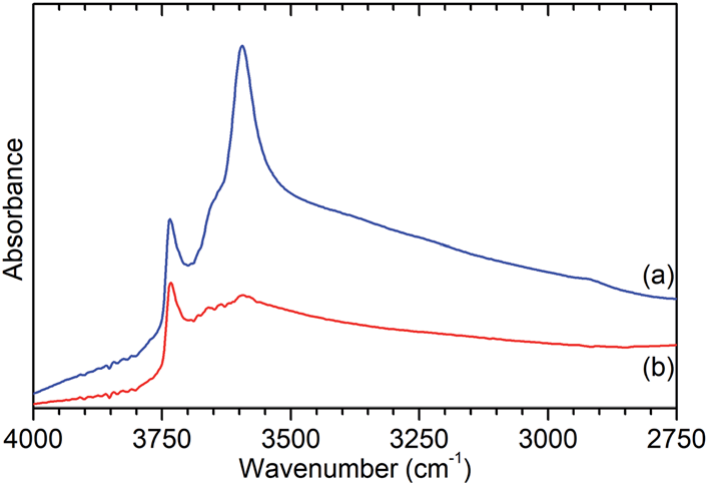
O–H stretch region of the infrared spectra of ZSM-5-FR (a) and ZSM-5-ST (b) recorded by DRIFTS. Spectral intensities normalised with respect to the silanol framework peak at 1875 cm^−1^. Full spectrum available as Fig. S2.†

Further characterisation shows that this de-alumination has had the expected effect of significantly reducing the level of Brønsted acidity in ZSM-5-ST compared to that in ZSM-5-FR. Infrared analysis of the O–H stretches (Fig. 2) shows a large reduction in the peak at 3595 cm^−1^, assigned to the O–H stretch of the zeolite Brønsted acid groups, with a slightly smaller reduction in the signal for the terminal silanol O–H species on the exterior of the crystals at 3736 cm^−1^.^15^ The signal due to O–H groups associated with extra-framework aluminium (EFAl) species, visible as a shoulder to the Brønsted peak at 3655 cm^−1^ in the ZSM-5-FR spectrum and an overlapping peak at the same position for ZSM-5-ST does not change significantly between the samples indicating that the EFAl species generated do not contain a significant quantity of O–H groups. These observations, combined with those drawn from the NMR data, are consistent with results in the literature which report that the pore structure of ZSM-5 favours the agglomeration of released aluminium into polymeric aluminium species rather than the octahedral aluminium-oxy-hydrides which make a significant contribution in steam-treated large pore zeolites such as zeolite HY.^18^

**Fig. 3:**
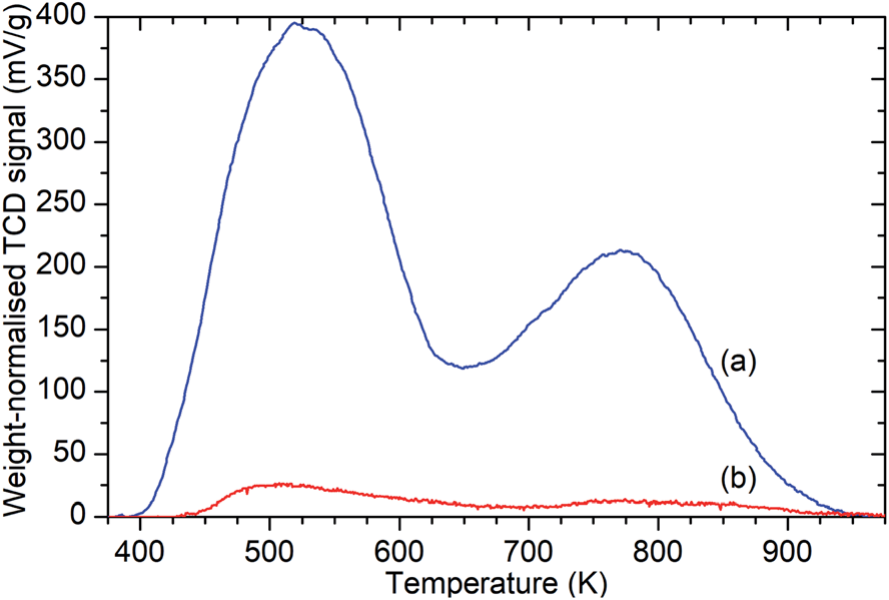
Weight-normalised ammonia desorption *vs.* temperature traces for ZSM-5-FR (a) and ZSM-5-ST (b) showing reduction in the number of both types of acid sites due to steam treatment. Peaks represent the desorption of ammonia molecules chemisorbed to silanol groups from 400–600 K and those bound to Brønsted acid sites from 650–900 K.^46^

Ammonia TPD analysis of the samples confirms the results observed in the infrared data, with significant reductions in the population of both Brønsted acid and silanol –OH groups. Fig. 3 shows the weight-normalised traces for the desorption of chemisorbed ammonia from saturated samples of ZSM-5-FR and ZSM-5-ST with temperature. Both samples show the release of two distinct populations of ammonia, assigned to molecules chemisorbed to silanol and Brønsted –OH groups.^46^ Normalisation of the integrated peak areas of these signals against those recorded for ammonia injections of known quantity allows the derivation of the exact levels of adsorbed ammonia in each environment and thus an accurate count of the acid groups, reproduced in Table 1. The final effect of the steam treatment is a 95% reduction in the acid site population in ZSM-5-ST relative to ZSM-5-FR and a 92% reduction in the number of silanol groups which are active for ammonia chemisorption. It is notable that this reduction in acid sites is considerably higher than the value for the loss of framework aluminium derived from the NMR data. Examination of the shape of the ammonia desorption curves shows that the maximum of the desorption peak assigned to the Brønsted groups lies at 770 K in both cases and that both samples retain chemisorbed ammonia at temperatures up to 950 K, indicating that the peaks represent ammonia interacting with the same type of acid groups in both samples and that neither of the peaks in the steamed sample are due to adsorption on EFAl species.

In contrast to the extensive chemical changes, there is little evidence of changes to the zeolite structure due to the steam treatment. Powder XRD examination of the two samples exhibits some minor broadening of the diffraction peaks, shown in Fig. S1,† but no significant changes in bulk structure. BET gas adsorption experiments using N_2_ as the adsorbant gas reveal that both the overall surface area and the proportion of that surface area which represents micropore channels of <3.5 Å diameter remain identical between samples within the degree of accuracy available (Table 1). Analysis of pore size distributions is of limited utility due to the limitations of analysing microporous materials with N_2_ as the adsorbent gas, however the distribution of the sample mesoporosity also does not appear to change significantly between samples. Taken together, these results indicate that the zeolite framework, and thus the overall microporous structure, remains intact despite the removal of the majority of the aluminium since these represent only ∼2.1% of the framework T-atoms. The broadening of the XRD pattern indicates that the removal of aluminium sites occurs essentially at random, increasing the amount of noise in the diffraction pattern. The increased mesoporosity in the BET results is due to the removal of framework atoms increasing the proportion of the voids which are greater than 3.5 Å in diameter.

**Fig. 4:**
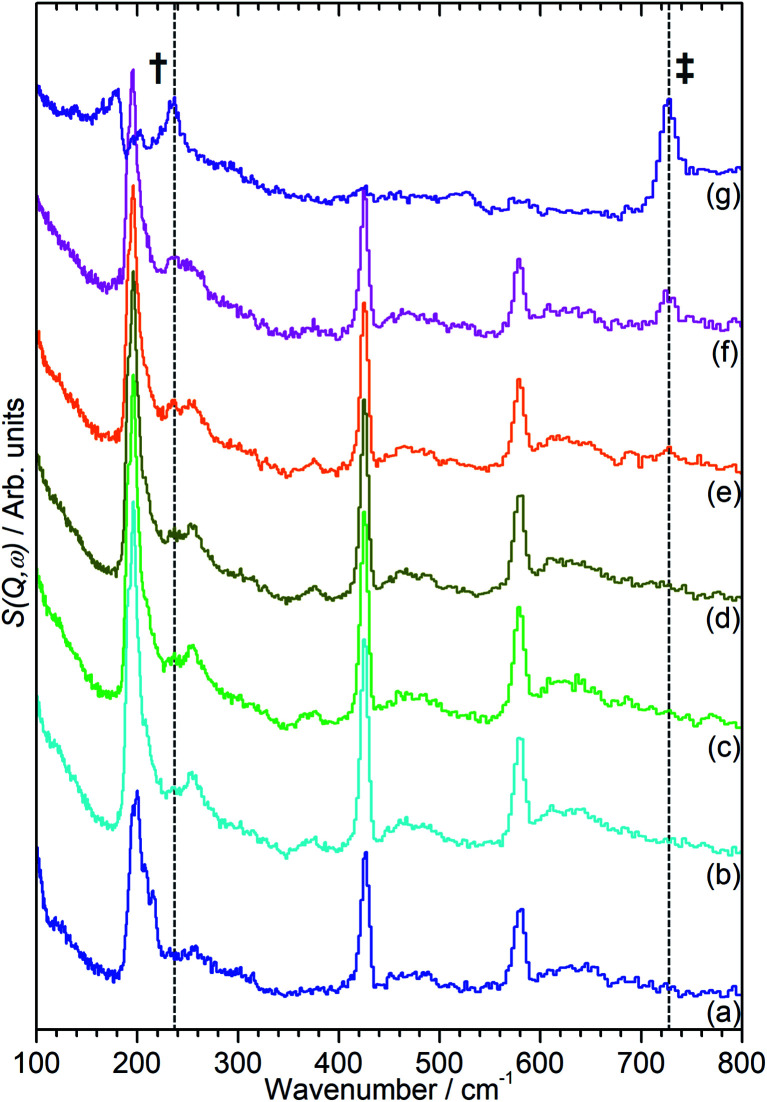
100–800 cm^−1^ region of the INS spectra of propene in ZSM-5-ST after adsorption at 170 K (a) then following further heating to: 260 K (b), 270 K (c), 280 K (d), 290 K (e), 300 K (f) and 325 K (g). The contributions of the zeolite framework have been subtracted from all spectra to render the changes in hydrocarbon modes more readily apparent and the spectra offset in the *y*-axis for clarity. The positions of the methyl torsion (†) and (–CH_2_–) in-phase rock (‡) in the oligomer spectrum at 325 K are highlighted.

The INS data collected allows us to follow the oligomerization reaction of propene within ZSM-5-ST in progress through observation of changes in the hydrocarbon deformation modes of the adsorbed species. The key changes are visible in the 100–800 cm^−1^ region of the spectra which are presented in Fig. 4 following subtraction of the unloaded zeolite spectrum to leave only the hydrocarbon contributions; the spectra across the full 0–1600 cm^−1^ region of optimal resolution on TOSCA, together with the unsubtracted data, is available in the ESI (Fig. S3 and S4).† The 100–800 cm^−1^ region contains the methyl torsion (200 cm^−1^), vinyl scissors (425 cm^−1^) and vinyl torsion (580 cm^−1^) modes of the pre-reaction physisorbed propene, while the formation of a new peak at 728 cm^−1^, assigned to the in-phase –CH_2_– rocking mode, and the shift of the methyl torsion to a new position at 235 cm^−1^ is characteristic of oligomer formation. These assignments are based on previous experimental studies that are backed up by theoretical calculations.^48^

It is immediately apparent from Fig. 4 that the reduction in acidity observed in the characterisation of ZSM-5-ST has had the expected effect of reducing its ability to catalyse olefin oligomerization at low temperatures. Whereas ZSM-5-FR can catalyse such reactions at temperatures as low as 225 K, with temperatures above 270 K resulting in oligomerization occurring fast enough that only the final product is observable if propene is introduced at this temperature,^42,48^ in ZSM-5-ST the first unambiguous sign of oligomerization activity is not observed until the sample has been heated to 290 K, as signified by the in-phase rock mode of the oligomer rising above the level of background noise. The continued presence of weak contributions from unreacted propene in Fig. 4(g) suggests that the reaction remains incomplete even at the highest temperature investigated by INS (325 K). The conclusion drawn is that the reduced acidity of ZSM-5-ST means that higher energies are required to initiate the protonation of the propene, which is believed to be the process that represents the rate-limiting step in the oligomerization reaction.^20,53^

**Fig. 5:**
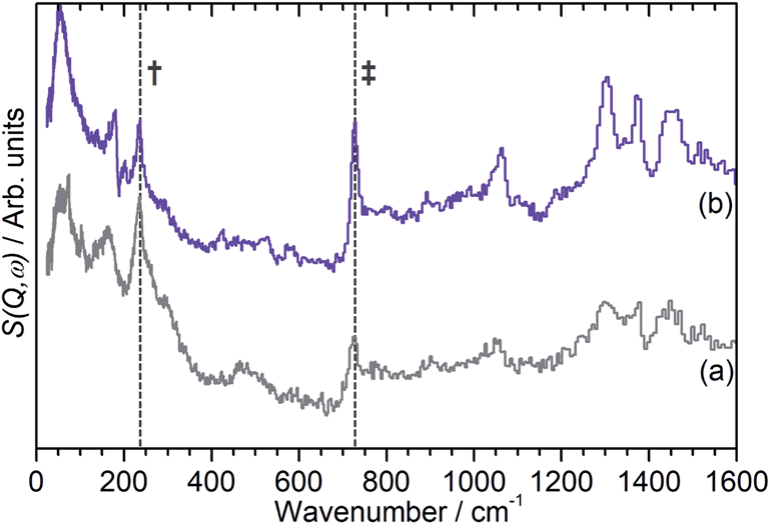
INS spectra of the final oligomer product of propene in ZSM-5-FR (a) and ZSM-5-ST (b) showing evidence of increased average chain length in ZSM-5-ST. Both spectra have had the zeolite contributions to the overall spectrum removed and the spectrum scaled to correct for the differing number of moles of propene in each sample. Positions of methyl torsion (†) and (–CH_2_–) in-phase rock (‡) highlighted. Spectra offset in *y*-axis.

Fig. 4 also exhibits some differences to the behaviour previously observed for propene oligomerization in the fresh catalyst prior to the initiation of the final oligomer formation. In ZSM-5-FR the formation of a hydrogen bonded propene intermediate as the first stage of the oligomerization reaction is visible as a splitting of the propene vinyl torsion peak into separate peaks for H-bonded and free propene at temperatures as low as 40 K below the temperature required for propene protonation and oligomerization.^48^ This intermediate is not observed in this case; since the reaction mechanism of the oligomerization in unlikely to have been affected, the most probable explanation is that the reduced number of acid sites means that the population of H-bonded propene in ZSM-5-ST is insufficient for the bonded species to be visible in the INS spectrum even when all acid sites are occupied. These differences in reaction behaviour result in slight differences in the composition of the product oligomer in each zeolite. As shown in Fig. 5, although in both cases the final product spectrum is characteristic of the formation of a primarily linear series of oligomer chains, evidenced by the lack of modes attributable to the presence of side chains and the formation of a broad band of overlapping methylene modes from 700–1100 cm^−1^, the spectra exhibit different relative intensities for the peaks in question. In particular, the reduced relative intensity of the methyl torsion mode and increased contribution from the in-phase methylene rock at 728 cm^−1^ in the ZSM-5-ST oligomer spectrum is evidence of a greater average chain length than in ZSM-5-FR. This is consistent with our previous hypothesis that the average oligomer chain length is determined by steric effects causing termination of the chain formation reaction, determined by the fact that the final product spectrum of propene oligomerizations in ZSM-5 is not temperature dependant. Since the protonated end of the oligomer is fixed in place at the location of the catalytic site it is the other end of the molecule which moves through the pore network as the chain is extended by propene additions. For the majority of forming oligomers at interior sites the limitation on maximum length will occur when this end of the chain encounters a pore channel which is already occupied by another molecule, preventing further growth since the oligomer has no further room for expansion. Since ZSM-5-ST has fewer, more widely spaced active sites the forming oligomer can extend further before intersecting another oligomer and having its growth blocked; resulting in the observed increase in average chain length.

In our previous studies of the reaction of propene with ZSM-5-FR the onset of the zeolite's ability to catalyse propene oligomerization has also been visible in the QENS data.^48^ Initiation of the oligomerization reaction results in the production of larger, less mobile species which temporarily reverses the trend of increased mobility with temperature which occurs as the sample warms. Since in QENS spectra the amount of movement in the sample determines the degree of inelastic broadening of the elastic peak the relative overall mobility of the sample at a given temperature can be quantified by determining the intensity of the elastic scattering at each temperature and normalising that value against the intensity at 20 K where it is assumed that the entire sample is immobile and all scattering intensity is elastic.^41^ This provides a measure of relative overall mobility *versus* temperature referred to as an elastic window (elwin) scan, which can be collected with even quite short measurement times at each point due to using the sum of scattered intensity across all scattering angles.

**Fig. 6:**
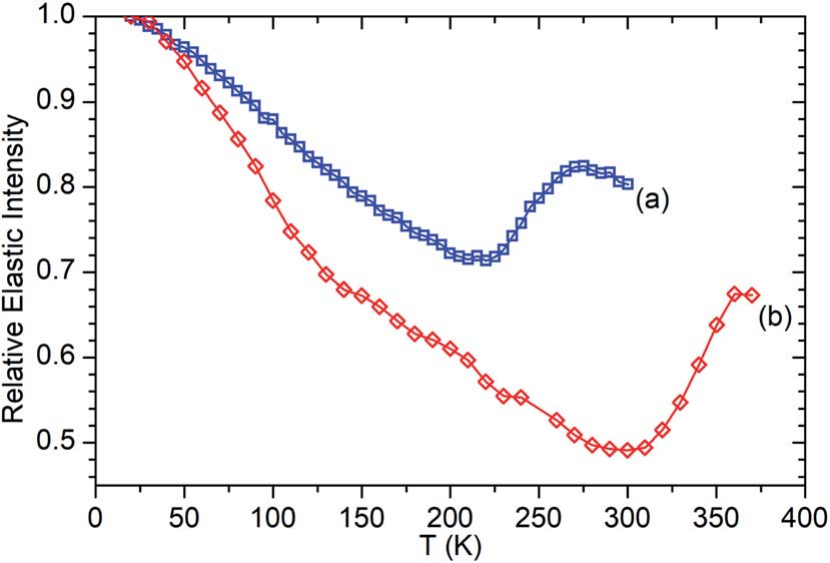
Relative elastic intensity *vs.* temperature for propene in ZSM-5-FR 20–300 K (a) and ZSM-5-ST from 20–370 K (b). Intensity values normalised against *T* = 20 K in both cases. ZSM-5-FR data reproduced from ref. 48.

Fig. 6 presents the elastic window scan collected for propene in ZSM-5-ST compared against the corresponding data for propene in ZSM-5-FR previously reported in ref. 48. The results confirm the observations about oligomerization requiring higher temperatures for initiation which were drawn from the INS dataset. As seen in the INS, the first signs of changes due to oligomerization are observed at 290 K with the deviation of the elastic window trace from the linear decrease with temperature observed from 130–280 K. Due to the shorter data collection time at each temperature allowing continuation of the testing to higher values we can also confirm the tentative identification of the continued presence of unreacted propene in the INS trace at 325 K since Fig. 6 shows that the system does not achieve its final state with all propene converted until 360 K. In addition to this decreased reactivity, the elastic window plot also allows some examination to be made of the effect of the steam treatment on the overall mobility of the propene in the two zeolites; most prominently that the ZSM-5-ST sample shows considerably higher mobility even at temperatures below 200 K where the effect of oligomerization does not contribute in either sample. Most of this difference is due to a more rapid increase in mobility in ZSM-5-ST from 20–140 K, at which point there is a visible inflection in the ZSM-5-ST elastic window trace above which the rate of mobility increase in each sample appears similar.

The linear nature of the ZSM-5-ST elastic window plot from 130–280 K suggests that the adsorbed propene is undergoing similar motions across this range and provides the opportunity to characterise these motions in more detail by means of the high resolution spectra collected at 170, 220 and 270 K. Comparison of the QENS spectra of the loaded and unloaded zeolite at these temperatures (Fig. S5†) shows increased scattering intensity for the loaded sample across the energy transfer range. The increased intensity in the elastic peak (*ω* = 0) is due to scattering from immobile propene and the intensity in the spectral wings (*ω* ≥ 0.4 meV) is due to scattering from propene moving so fast that it is outside the dynamic window of the OSIRIS spectrometer. The increased broadening of the central peak in all three loaded samples relative to their unloaded counterparts indicates that there is motion occurring within the dynamic window and which is therefore amenable to fitting analysis.

**Fig. 7:**
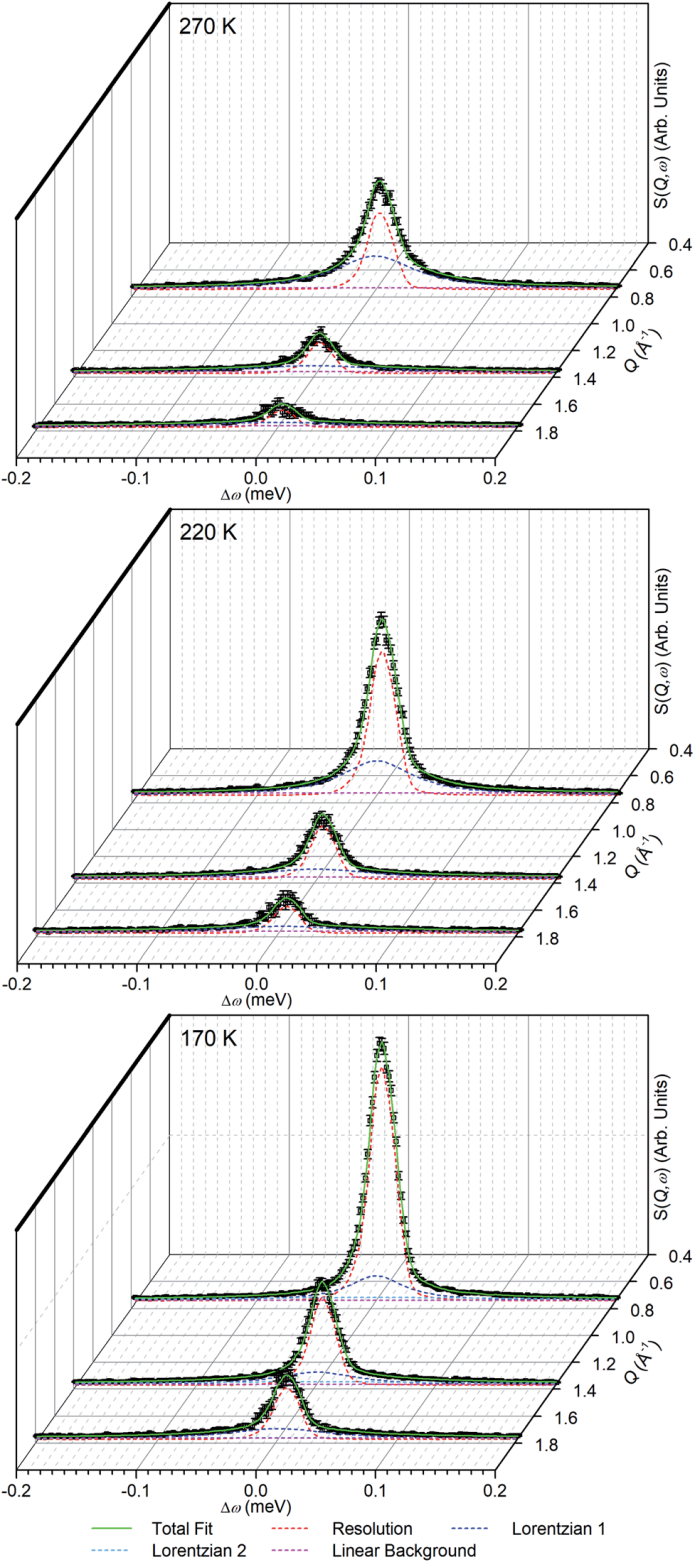
Examples of fits to the experimental data obtained for propene in ZSM-5-ST at the indicated temperatures. Elastic scattering is modelled by a scaled instrumental resolution function, with Lorentzian functions and the linear background representing motions occurring inside and outside the instrument resolution window respectively.

In order to simplify the fitting process, the collected spectra for the unloaded sample at each temperature were subtracted from the corresponding loaded sample in order to eliminate scattering from the sample cell and zeolite framework, leaving only the propene contributions. These were then fitted to a convolution of a delta function with the instrument resolution function, a flat background and quasielastic terms according to eqn (1) in order to extract the quasielastic component(s) as a function of *Q*. The instrument resolution function was provided by the base temperature spectrum of the loaded sample that was recorded for this purpose. For the 220 and 270 K spectra the quasielastic component was adequately described by a single Lorentzian, corresponding to a single motion occurring within the resolvable time window. For the 170 K dataset this model did not produce an acceptably close fit to the experimental data and better results were obtained by use of two Lorentzian functions; one with similar *Q*-dependant behaviour to that observed at higher temperatures and the second considerably broader. Examples of the fits obtained at each temperature are shown in Fig. 7 for selected *Q* values.

**Fig. 8:**
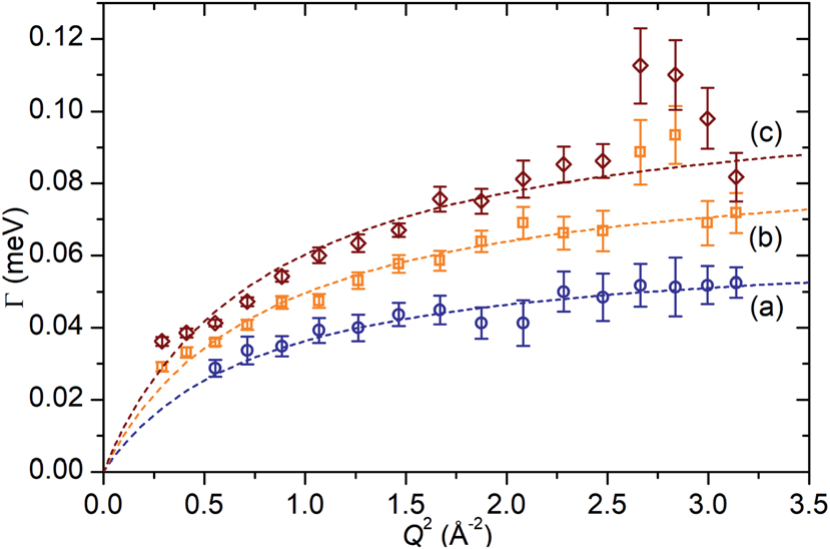
Variation of Lorentzian linewidth (*Γ*) as a function of *Q*^2^ for propene in ZSM-5-ST at 170 K (a), 220 K (b) and 270 K (c). Dotted lines represent best fit of the data to the Singwi–Sjölander model using the parameters given in Table 2.

**Fig. 9:**
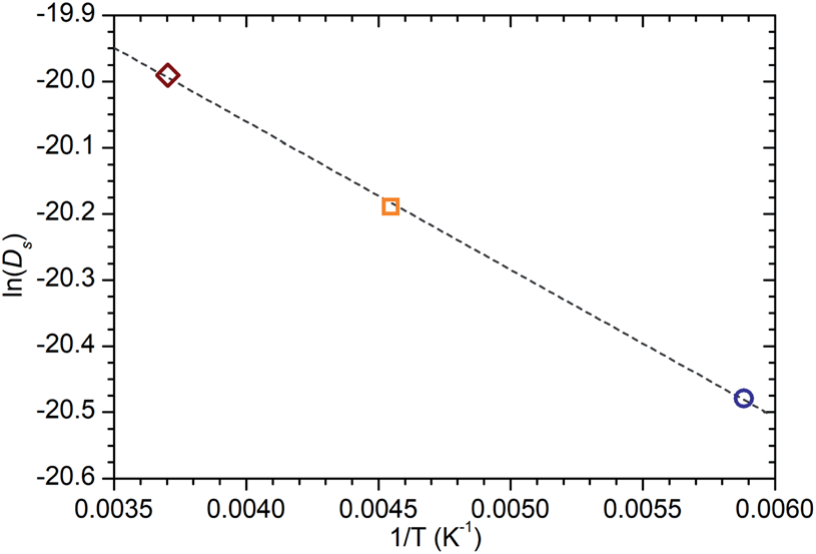
Arrhenius plot of the self-diffusion coefficients of propene in ZSM-5-ST at 170, 220 and 270 K calculated from QENS data collected at these temperatures showing a linear relationship across this temperature range. Line of best fit to the experimental data shown as the dotted line, giving an activation energy of 1.86 kJ mol^−1^ for this motion.

In the case of the Lorentzian which is observed at all temperatures, Fig. 8 shows the variation of its HWHM as a function of *Q*^2^ showing similar behaviour at all three temperatures. The lower energy resolution limit of OSIRIS at 25.4 μeV means that the Lorentzian is indistinguishable from the elastic peak below 0.5 Å^−2^ at 170 K and below 0.3 Å^−2^ at any temperature resulting in no HWHM points being available below these values. The anomalous results for the 220 and 270 K data in the region 2.6 Å^−2^ ≤ *Q*^2^ ≤ 2.9 Å^−2^ are due to the presence of a Bragg peak from the zeolite framework at these scattering angles which increases the intensity of the elastic contributions to the *S*(*Q*,*ω*) function at this position and results in worse signal : noise levels in the propene-only function once the immobile contributions are subtracted. The type of non-linear *Q*^2^ dependence observed here is characteristic of broadening due to jump diffusion, with the present case most closely corresponding to the model of Singwi and Sjölander; this proposes a system where the scatterers perform periodic jumps between low-energy positions but are also able to oscillate around those positions between jumps, with the peak width variation given by:2
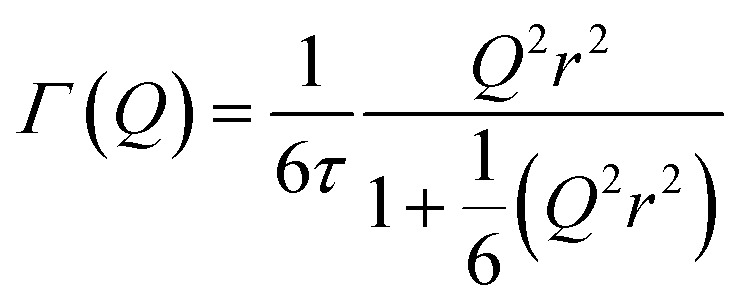
where *τ* is the residence time between jumps and 〈*r*^2^〉 the mean square jump length.^54^ A least-squares fit of the data to this equation gives the traces shown as the dotted lines in Fig. 8. Table 2 presents the derived values for *τ* and 〈*r*^2^〉, together with calculated values for the self-diffusion coefficient which can be derived from the model parameters by the relationship *D*_s_ = 〈*r*^2^〉/6*τ*. The differences in jump length distribution may be regarded as being within the level of experimental error in the fit analysis, meaning that the process which is being modelled by this Lorentzian corresponds to propene diffusing through jumps of approximately 2.8 Å length with the time between jumps decreasing with temperature. This jump length indicates that the jumps in this motion are not occurring between pore intersections in the zeolite framework, which are separated by 9 Å in the straight [010] channels and 12 Å in the [100] channels but rather it represents movements on a shorter length scale. Since the distance is also less than the diameter of the 10-ring framework window which forms the limiting radius of the zeolite pores (5.1 × 5.5 Å in the narrower sinusoidal channel), it is likely that propene–propene interactions play an important role in determining the favoured jump length. The values for the diffusion coefficients follow a linear Arrhenius relationship across the temperature range investigated (Fig. 9) allowing an estimate of 1.86 kJ mol^−1^ to be made for the activation energy of this motion.

**Table 2:** Summary of diffusion parameters derived from QENS data fitting for propene in ZSM-5-ST

*T* (K)	*τ* (ps)	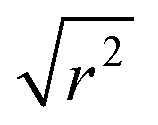 (Å)	*D*_s_ (m^2^ s^−1^)
170	10.27	2.80	1.28 × 10^−9^
220	7.38	2.75	1.71 × 10^−9^
270	6.10	2.76	2.08 × 10^−9^

While information on the diffusion behaviour of olefins in zeolites is scarce, the use of the technique to study movement of alkanes in similar environments is well established and provides some points of useful comparison with the results obtained here. Using the Arrhenius relationship described above allows the diffusion constant of propene in ZSM-5-ST at 300 K to be estimated as being 2.3 × 10^−9^ m^2^ s^−1^. As shown in Table 3, this is approximately 45% of the value for the self-diffusion of propane in an unsteamed H-ZSM-5 determined by Silverwood and Sakai^39^ at the same temperature. Since the H-ZSM-5 used in that study is essentially identical to the ZSM-5-FR used as the starting material here, this difference in diffusion constant is attributable to the greater rigidity of propene compared with propane and stronger zeolite–adsorbate interactions due to the double bond in propene creating greater energy barriers to diffusion. Since the estimated jump length for the movement of propane is similar to that obtained here, it is likely that the increased interaction strength is the greater contributor; this also provides further evidence that the micropore structure of ZSM-5-ST is similar to its pre-treatment counterpart. These comparisons are complicated by the fact that diffusion constants are affected by the level of gas loading in the zeolite,^33^ and the propane study was conducted at much higher gas loadings than are the case in this study. A study by Jobic, *et al.*^55^ provides a closer match in terms of gas loading level (4 molecules per unit cell) but was conducted using Na-ZSM-5, and the larger size of the Na counter ion in this material likely explains the fact that the diffusion reported in this case is 45% slower than our observed propene diffusion and occurs over considerably longer jump distances. Despite these limitations, the results of all these studies are within an order of magnitude, providing evidence that we are observing a similar motion in all three cases.

**Table 3:** Comparison of estimated diffusion constant of propene in ZSM-5-ST at 300 K with literature values for the diffusion of propane in H-ZSM-5 (ref. 39) and Na-ZSM-5 (ref. 55) at the same temperature

Material	Adsorbate	Loading (molecule per u.c.)	*τ* (ps)	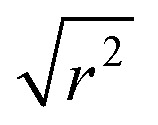 (Å)	*D*_s_ (m^2^ s^−1^)
ZSM-5-ST	Propene	5.06	—	—	2.3 × 10^−9^
H-ZSM-5	Propane	Unknown	3.0	2.86	5.2 × 10^−9^
Na-ZSM-5	Propane	4	90	8.4	1.3 × 10^−9^

The diffusion constants are also consistent with those predicted by molecular dynamics (MD) simulations of small molecules in MFI framework structures. Nowak, *et al.*^56^ predict a *D*_s_ of 4.1 × 10^−9^ m^2^ s^−1^ for propane in silicalite (the pure SiO_2_ analogue of ZSM-5) at a loading of 8 molecules per unit cell. The work of Leroy and co-workers^57^ establishes a relationship between diffusivity and alkane chain length which also predicts a diffusion constant of approximately 4 × 10^−9^ m^2^ s^−1^ for propane at a 4 molecules per unit cell loading level, although propane was not simulated directly in that study. Although these simulations make a large number of simplifying assumptions to aid computation, including the use of purely silicaceous frameworks without defects, the similarity in diffusion constants is interesting because in the case of these MD simulations it is calculated from the mean squared displacements of the propane molecule over time scales of up to one nanosecond and therefore represents the diffusion responsible for its transport through the zeolite. The similar *D*_s_ values mean that both the QENS and MD results considered here probably represent the same motion. From the combination of these we can therefore deduce that in ZSM-5 zeolites both propane and propene diffuse through the structure by means of jumps of approximately 3 Å length between low energy sites. Residence time within jumps is determined by the strength of interaction between the adsorbate and the zeolite, resulting in propene having a longer residence time and lower diffusion constant than propane. Similarly, the reduction in the number of acid sites in ZSM-5-ST results in weaker zeolite–propene interactions than is the case in ZSM-5-FR when the sample as a whole is considered, resulting in shorter average residence times in ZSM-5-ST and producing the increased overall mobility observed in Fig. 6.

**Fig. 10:**
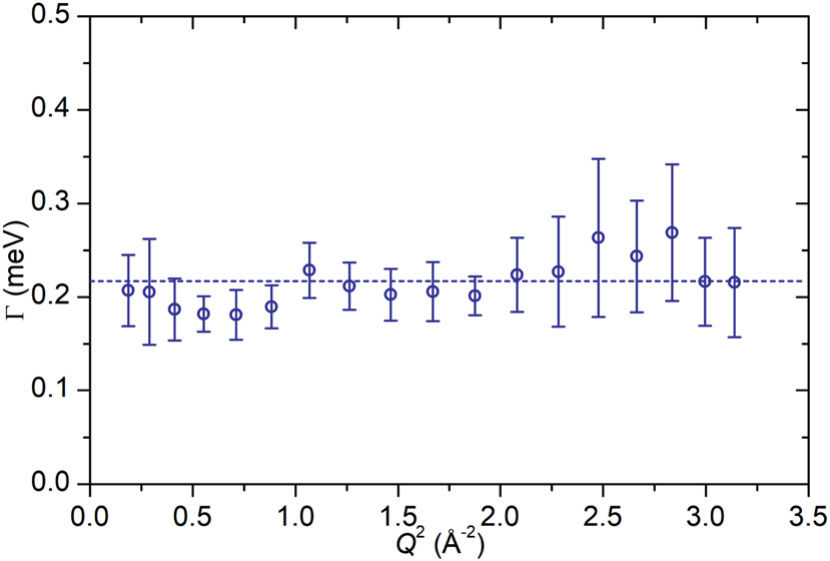
Variation of Lorentzian linewidth (*Γ*) as a function of *Q*^2^ for the second Lorentzian observed for propene in ZSM-5-ST at 170 K showing *Q*-independent behaviour. Dotted line represents the line of best fit.

In contrast to the first Lorentzian, the second Lorentzian, which is only observed at 170 K, is considerably broader. This leads to some difficulty in separating its contribution from the linear background resulting in the larger degree of uncertainty in the *Γ vs. Q*^2^ plot shown in Fig. 10, although the greater magnitude of the energy transfer values in this case means that this Lorentzian remains resolvable across the full *Q* range accessible on the instrument. Within the limits of experimental accuracy, the *Γ* values appear constant and independent of *Q*, indicative of this Lorentzian corresponding to a rotational movement of the propene. The line of best fit to the experimental values provides an estimate of the rotational constant for the motion of *D*_r_ = 3.29 × 10^11^ s^−1^. Due to this Lorentzian not being observable at higher temperatures, we can surmise that it represents a rapid rotation of the propene molecule which is only observable at extremely low energies, and at higher temperatures moves quickly enough that it is outside the instrumental dynamic window and so merges into the flat background. This rapidity also explains why similar motions have not been reported in other studies in the literature, which generally only consider higher temperatures.

## Conclusions

These results show that the de-alumination of a H-ZSM-5 zeolite has considerable effect on its interactions with reactive olefin species, such as propene, in terms of acid–olefin bonding and reactivity. The reduction in overall framework acidity results in higher temperatures being required to H-bond and protonate the propene molecule to initiate the oligomerization reaction. This is even though characterisation of the acid groups by ammonia TPD suggests that the individual acid sites have the same strength in both the steamed and unsteamed material, with only their population within the zeolite being affected. Since the initial protonation is believed to be the rate-limiting step in olefin oligomerization,^20,53^ the reaction appears to propagate at a similar rate to that observed for ZSM-5-FR, once the higher energies required to initiate it in ZSM-5-ST are reached. However, the product oligomer mix in ZSM-5-ST shows evidence of a longer average chain length due to the greater separation of active sites resulting in less steric restriction of maximum chain length due to intersection of the growing oligomers.

QENS analysis allows the movement of propene in the steamed ZSM-5 to be probed on a molecular scale and suggests that the propene diffuses through the pore structure by means of a jump diffusion mechanism. The rate of this motion varies from 1.3–2.1 × 10^−9^ m^2^ s^−1^ depending on temperature and occurs with a mean jump distance of approximately 3 Å regardless of temperature, indicating that the geometry of the motion is dictated by the constraints of the zeolite pore network. These diffusion rates are comparable to those reported in the literature for propane in similar systems with propene diffusing at approximately half the speed of propane but with the average jump distance being essentially identical, indicating that stronger zeolite–adsorbate interactions due to the C=C bond result in the propene spending longer oscillating in its low energy resting positions between jumps. Comparison with results obtained for propene in ZSM-5-FR shows the de-alumination has had the effect of increasing mobility within the zeolite, likely due to reducing the number of propene groups immobilised at acid sites.

The differences in behaviour observed here illustrate the important distinction between the reactivity of fresh acid zeolites and that of materials which are more representative of catalysts in industrial use, especially once they have reached steady-state operating conditions. The conditions probed here are relatively mild and only concern one aspect of the reactions of olefins over acid zeolites; differences in behaviour are likely to become even more pronounced at the higher temperatures associated with cracking and isomerisation reactions. Since the energy transfer associated with the jump diffusion lies within the dynamic window accessible on existing spectrometers, QENS techniques are well positioned to obtain molecular diffusion data for adsorbed olefins close to process conditions.

## Author contributions

A. P. Hawkins (neutron data acquisition, manuscript original draft, sample characterisation), A. Zachariou (neutron data acquisition, sample characterisation), S. F. Parker (management of neutron experiments, manuscript construction), P. Collier (conceptualization), N. Barrow (NMR spectroscopy), I. P. Silverwood (QENS), R. F. Howe (validation, manuscript construction), D. Lennon (project management, supervision, manuscript construction).

## Conflicts of interest

There are no conflicts to declare.

## Supplementary Material

RA-010-D0RA03871G-s001
