# Supplementary material for: Effect of steam de-alumination on the interactions of propene with H-ZSM-5 zeolites
Source: RSC Adv. 2020 Jun 17;10(39):23136–47. doi: 10.1039/d0ra03871g (PMC9054920; doi:10.1039/d0ra03871g)
Supplement: RA-010-D0RA03871G-s001 [file RA-010-D0RA03871G-s001.pdf]

Electronic Supplementary Material (ESI) for RSC Advances.
This journal is © The Royal Society of Chemistry 2020

Effect of steam de-alumination on the interactions of propene with H-ZSM-5 zeolites.

Electronic Supplementary Information.

Alexander P. Hawkins,[ab] Andrea Zachariou,[ab] Stewart F. Parker,[abc] Paul Collier,[d] Nathan Barrow,[d] Ian P. Silverwood,[c] Russell F. Howe,[e] and David Lennon*[ab]

[a] School of Chemistry, University of Glasgow, Joseph Black Building, Glasgow G12 8QQ, UK

[b] UK Catalysis Hub, Research Complex at Harwell, STFC Rutherford Appleton Laboratory, Chilton, Oxon OX11 0FA, UK

[c] ISIS Neutron and Muon Source, STFC Rutherford Appleton Laboratory, Chilton, Oxon OX11 0QX, UK

[d] Johnson Matthey Technology Centre, Blounts Court, Sonning Common, Reading RG4 9NH, UK

[e] Department of Chemistry, University of Aberdeen, Aberdeen, AB24 3UE, UK

Sample:	Fit Parameters								
	Peak 1 (AlO_4)			Peak 2 (AlO_6)			Peak 3 (Intermediate species)		
	Centre (ppm)	FWHM (ppm)	Relative Area	Centre (ppm)	FWHM (ppm)	Relative Area	Centre (ppm)	FWHM (ppm)	Relative Area
**ZSM-5-FR**	53.4	5.1	1.000	-1.8	5.1	0.080	32.7	7.1	0.017
**ZSM-5-ST**	54.5	12.8	0.227	2.7	12.8	0.180	29.8	21.9	0.309

Table S1: Fit parameters for NMR spectra in Figure 1 used to derive relative intensities for each ^{27}Al environment.

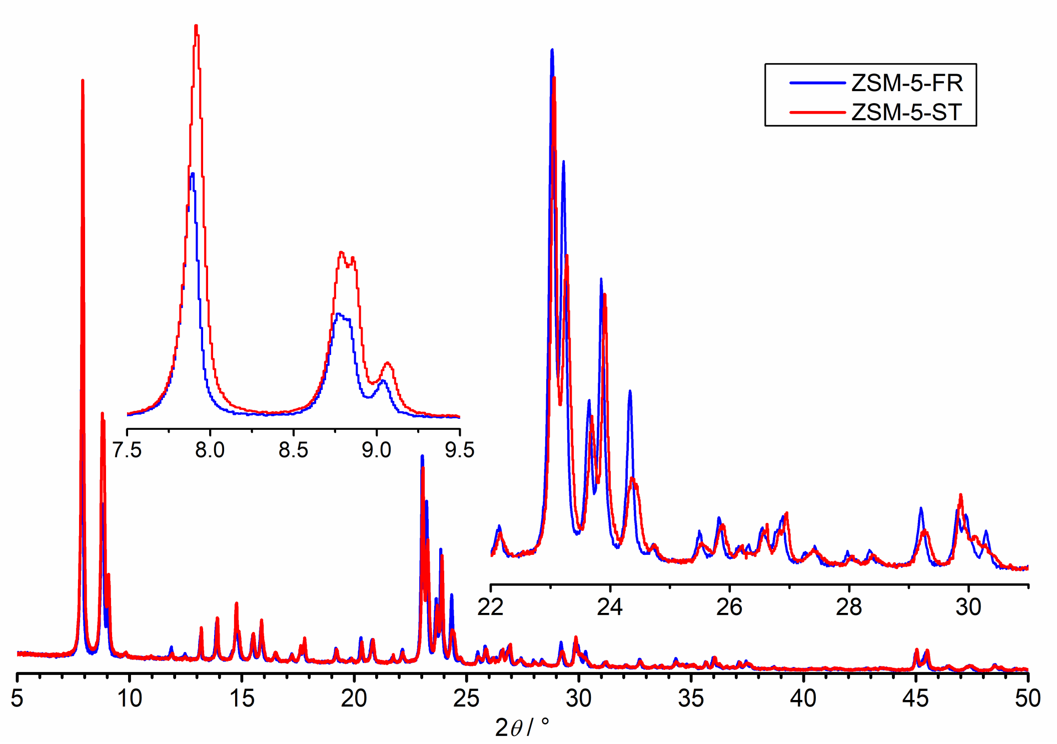

Figure S1: Powder XRD diffraction pattern of ZSM-5 before and after steam treatment.

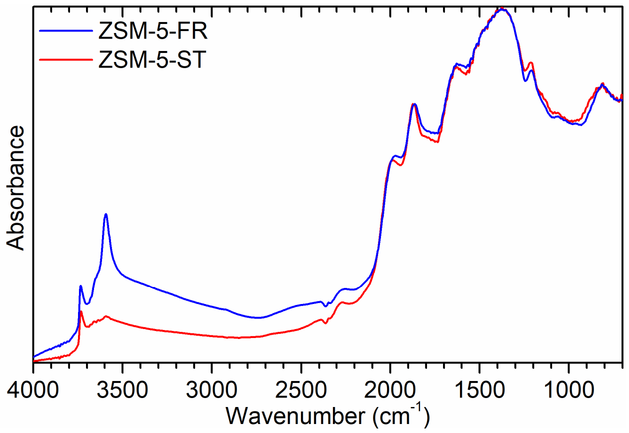

Figure S2: Infrared spectra of ZSM-5-FR and ZSM-5-ST recorded by DRIFTS. Spectral intensities normalised with respect to the silanol framework peak at 1875 cm^{-1}.

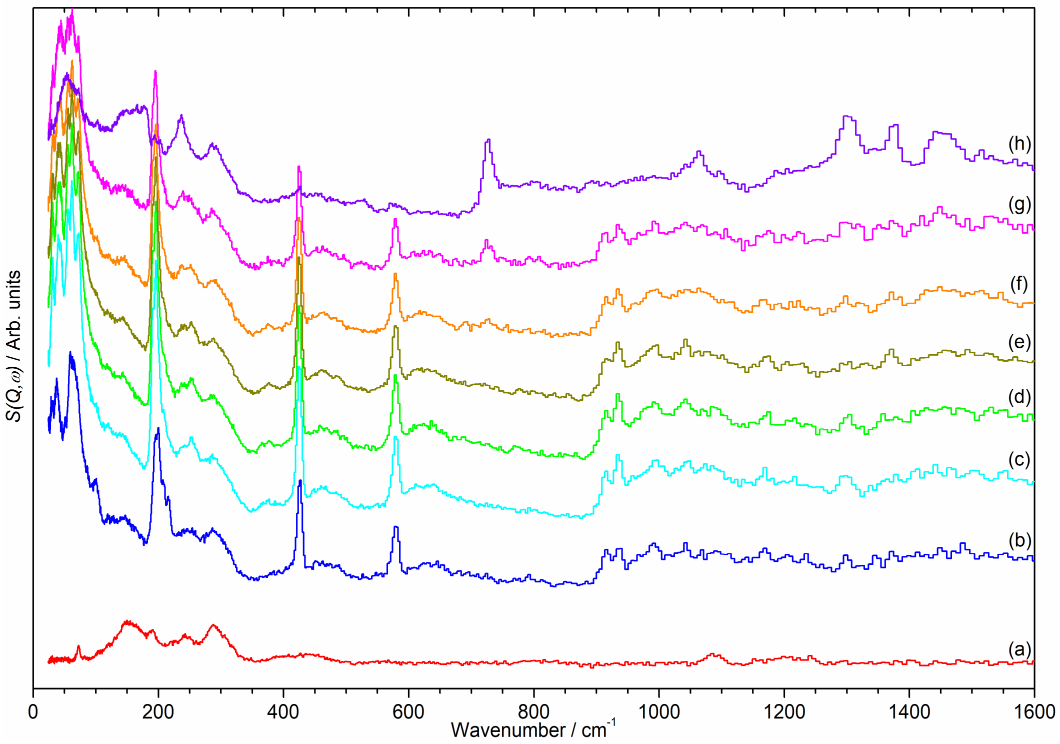

Figure S3: Full INS spectra of ZSM-5-ST before (a) and after (b) adsorption of propene at 170 K then following further heating to: 260 K (c), 270 K (d), 280 K (e), 290 K (f), 300 K (g) and 325 K (h). Spectra offset in the y-axis for clarity.

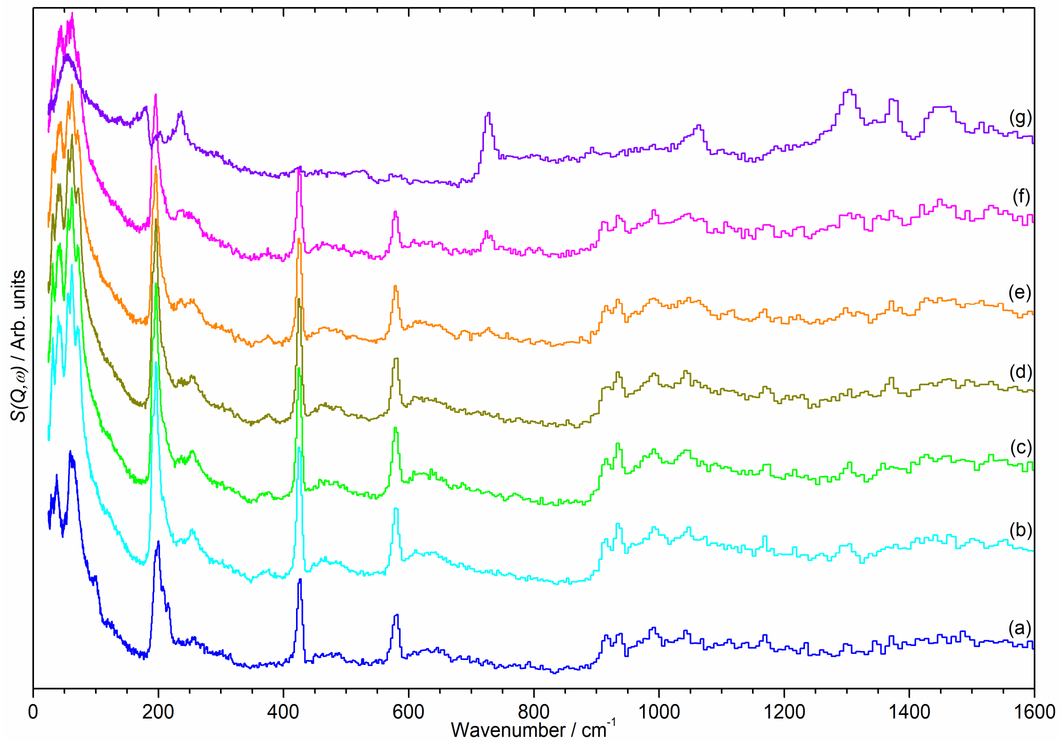

Figure S4: Propene-only component of the full INS spectra of propene in ZSM-5-ST after adsorption at 170 K (a) then following further heating to: 260 K (b), 270 K (c), 280 K (d), 290 K (e), 300 K (f) and 325 K (g) produced by subtracting the contributions of the zeolite framework (Figure S3a) from each subsequent spectrum (5b-5h). Spectra offset in the y-axis for clarity.

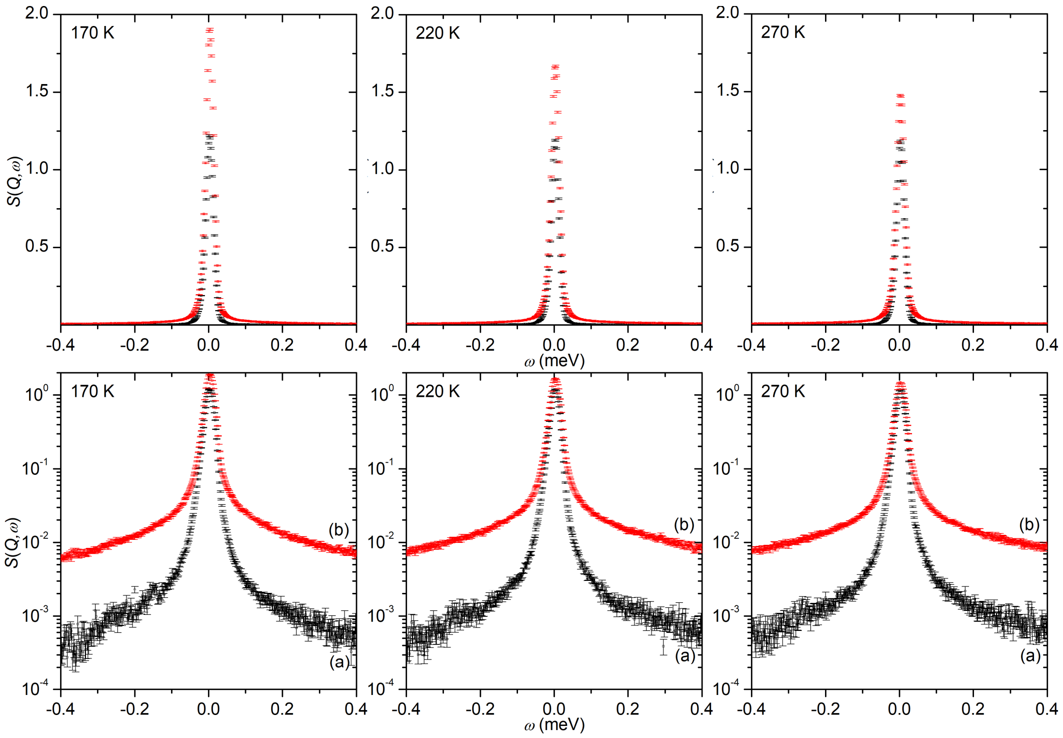

Figure S5: Comparison of quasielastic peaks for ZSM-5-ST (a) and propene in ZSM-5-ST (b) at 170, 220 and 270 K. Linear scale (top) shows increased intensity of the elastic scattering peak due to propene adsorption with the magnitude diminishing with temperature due to increased propene motions. Log scale (bottom) shows the form of these quasielastic motion as increased wing scattering intensity and quasielastic peak broadening. Peaks are the sum of scattered intensity across all values of Q to improve signal:noise levels.
